# Molecular Design of Bisphosphonate-Modified Proteins for Efficient Bone Targeting *In Vivo*

**DOI:** 10.1371/journal.pone.0135966

**Published:** 2015-08-19

**Authors:** Hidemasa Katsumi, Jun-ichi Sano, Makiya Nishikawa, Keiko Hanzawa, Toshiyasu Sakane, Akira Yamamoto

**Affiliations:** 1 Department of Biopharmaceutics, Kyoto Pharmaceutical University, Kyoto, Japan; 2 Department of Biopharmaceutics and Drug Metabolism, Graduate School of Pharmaceutical Sciences, Kyoto University, Kyoto, Japan; Baker IDI Heart and Diabetes Institute, AUSTRALIA

## Abstract

To establish a rational molecular design for bisphosphonate (BP)-modified proteins for efficient bone targeting, a pharmacokinetic study was performed using a series of alendronate (ALN), a nitrogen-containing BP, modified proteins with various molecular weights and varying degrees of modification. Four proteins with different molecular weight—yeast glutathione reductase (GR; MW: 112,000 Da), bovine serum albumin (BSA; MW: 67,000 Da), recombinant human superoxide dismutase (SOD; MW: 32,000 Da), and chicken egg white lysozyme (LZM; MW: 14,000 Da)—were modified with ALN to obtain ALN-modified proteins. Pharmacokinetic analysis of the tissue distribution of the ALN-modified and unmodified proteins was performed after radiolabeling them with indium-111 (^111^In) by using a bifunctional chelating agent. Calculation of tissue uptake clearances revealed that the bone uptake clearances of ^111^In-ALN-modified proteins were proportional to the degree of ALN modification. ^111^In-GR-ALN and BSA-ALN, the two high-molecular-weight proteins, efficiently accumulated in bones, regardless of the degree of ALN modification. Approximately 36 and 34% of the dose, respectively, was calculated to be delivered to the bones. In contrast, the maximum amounts taken up by bone were 18 and 13% of the dose for ^111^In-SOD-ALN(32) and LZM-ALN(9), respectively, because of their high renal clearance. ^111^In-SOD modified with both polyethylene glycol (PEG) and ALN (^111^In-PEG-SOD-ALN) was efficiently delivered to the bone. Approximately 36% of the dose was estimated to be delivered to the bones. In an experimental bone metastasis mouse model, treatment with PEG-SOD-ALN significantly reduced the number of tumor cells in the bone of the mice. These results indicate that the combination of PEG and ALN modification is a promising approach for efficient bone targeting of proteins with a high total-body clearance.

## Introduction

The prevalence of bone diseases is rapidly increasing as the population ages. Various endogenous proteins, including enzymes and cytokines, represent new candidates as therapeutic agents to treat bone diseases [1–4]. However, most protein drugs intended for bone diseases do not have a high affinity for bone hydroxyapatite under physiological conditions, which limits their therapeutic potential [1]. Therefore, targeting protein drugs to the bone is essential for the treatment of bone diseases. Of the various strategies available, chemical modification is an attractive approach for the targeted delivery of protein drugs, in terms of efficacy and safety, because protein drugs have several functional groups that can be conjugated with ligands or polymers [5].

To date, bone targeting of protein drugs by chemical modification has been studied with several molecules, including tetracycline, acidic oligopeptides, and bisphosphonates (BPs) [6–8]. BPs strongly inhibit the function of osteoclasts that mediate bone resorption, thereby allowing for their use in the treatment of Paget’s disease, bone metastasis, hypercalcemia of malignancy, and osteoporosis [9,10]. In addition, BPs are carbon-substituted pyrophosphate analogs that exhibit a high affinity for hydroxylapatite. Consequently, BPs are rapidly distributed to the bone or excreted in the urine in equal proportion following intravenous injection [11,12]. Accordingly, the modification of protein drugs with BPs could be useful for bone targeting. It was reported that the bone accumulation of osteoprotegerin, a potent receptor activator of nuclear factor κB ligand (RANKL) inhibitor that prevents osteoclastogenesis, was increased following BP modification [13]. Furthermore, bone metastasis was suppressed by intravenous injection of BP-modified bovine catalase in mice [14]. Despite these promising characteristics, there are no systemic studies on the application of BP modification to the bone targeting of protein drugs with different physicochemical and biological characteristics. In fact, the relationship between the physicochemical characteristics of proteins, such as the molecular weight and degree of modification with BP, and their *in vivo* pharmacokinetic profiles are not fully understood.

The aim of the present study was to establish a rational molecular design of BP-modified proteins for efficient bone targeting *in vivo*. In this study, we used the nitrogen-containing BP, alendronate (ALN) and conjugated it to various proteins to obtain ALN-modified proteins. The bone targeting efficiency of ALN-modified proteins with various molecular weights and degree of modification was examined after intravenous injection in mice, and pharmacokinetic analyses were performed to obtain quantitative relationships between these two factors.

## Materials and Methods

### Animals

Male ddY mice (5-week-old) and female C57/BL6 mice (7-week-old) were purchased from Japan SLC Inc. (Shizuoka, Japan), and were maintained under conventional housing conditions. All animal experiments were conducted in accordance with the principles and procedures outlined in the National Institutes of Health Guide for the Care and Use of Laboratory Animals. The Animal Experimentation Committee of the Kyoto Pharmaceutical University approved all protocols for animal experiments.

### Chemicals

Glutathione reductase from yeast (GR, 1,000 U/mL) was purchased from Oriental Yeast, Tokyo, Japan. Bovine serum albumin (BSA) was purchased from Sigma-Aldrich, St. Louis, MO. Human recombinant superoxide dismutase solution (SOD, 300 or 450 U/μL) was purchased from Wako Pure Chemical Industries, Osaka, Japan. Lysozyme from chicken egg whites (LZM, >9,000 U/mg) was purchased from MP Biomedicals, Illkirch, France. Alendronic acid, monosodium salt, trihydrate (ALN) was purchased from Toronto Research Chemicals, Brisbane, North York, Canada. Methoxypolyethylene glycol *N*-succinimidyl succinate (activated PEG; average molecular weight 5,200 Da) was purchased from NOF Co, Tokyo, Japan. 1-Ethyl-3-(3-dimethylaminopropyl)carbodiimide (EDAC), diethylenetriaminepentaacetic acid (DTPA) anhydride and 4-(2-hydroxyethyl)-1-piperazineethanesulfonic acid (HEPES) were purchased from Dojindo Laboratory, Kumamoto, Japan. D-luciferin was purchased from Promega, Madison, WI. Pharmalyte 3–10 and amberlyte IRN-150L were purchased from GE Healthcare UK Ltd.,Buckinghamshire, UK.^111^Indium chloride ([^111^In]InCl_3_) was supplied by Nihon Medi-Physic, Takarazuka, Japan. All other chemicals were obtained commercially as reagent-grade products.

### Synthesis and characterization of ALN-modified proteins

The ALN-modified proteins were synthesized by covalent attachment of ALN to the proteins. GR (MW: 112,000 Da), BSA (MW: 67,000 Da), SOD (MW: 32,000 Da) and LZM (MW: 14,000 Da) were used as model proteins in this study. The coupling reaction of a protein with amine of ALN by EDAC was performed according to a previously published method with slight modifications [15]. In brief, protein was added to the ALN dissolved in 0.1 M HEPES (pH 6.5) with EDAC as a carboxyl activating agent. After incubation on ice for 1 h, a second aliquot of EDAC was added to the solution, and the reaction mixture was stirred overnight at 4°C. The pH of the reaction solution was adjusted to 6.5–7.0 during the first 2 h. The number of ALN residues per protein molecule was controlled by the molar ratio of reagents, protein, ALN and EDAC. The reaction mixture was applied to a column (Sephadex G-25 coarse, GE Healthcare, Little Chalfont, UK) and fractions with larger molecular weights were collected. To remove protein aggregates formed during the synthesis, reaction products were ultrafiltered through a membrane (Vivaspin, GE Healthcare, Little Chalfont, UK) that traps and retains large protein aggregates. Each derivative was then washed and concentrated by ultrafiltration against distilled water, and lyophilized. These protein derivatives were subjected to SDS-PAGE analysis, and all were found to produce one band (data not shown). To synthesize the polyethylene glycol-SOD-ALN conjugate (PEG-SOD-ALN), SOD was mixed with activated PEG in borate buffer (pH 9.0) for 24 h at 4°C in the dark [16], and then mixed with ALN as described above. The number of ALN conjugated to each derivative was determined by the molybdenum method [17]. The number of PEG was estimated by the number of unreacted free amino groups, which was determined by the trinitrobenzenesulfonic acid method [18]. The apparent molecular weight of each derivative was estimated in reference to the number of ALN and PEG.

### Isoelectric point of native proteins and ALN-modified proteins

The isoelectric point (pI) was measured by isoelectric focusing as reported previously [19]. The gel consisted of acrylamide, bisacrylamide, glycerol, pharmalyte 3–10 for IEF, amberlyte IRN-150L, TEMED, and ammonium persulfate. H_2_SO_4_ (0.1 M) and NaOH (0.1 M) were used as the anolyte and catholyte, respectively. Samples were applied on the gel and subjected to electrophoresis at 500 V for 9 h, 600 V for 1 h and 800 V for 2 h. *Aspergillus niger* amyloglucosidase (pI 3.5), *Aspergillus niger* glucose oxidase (pI 4.2), soybean trypsin inhibitor (pI 4.5), bovine milk β-lactoglobulin (pI 5.3, 5.2), bovine erythrocytes carbonic anhydrase (pI 6.0), horse muscle myoglobin (pI 6.9, 7.4), *Lens culinaris* lectin (pI 7.8, 8.0, 8.3), bovine pancreas ribonuclease A (pI 9.5), and pig heart cytochrome C (pI 10.7) were used as pI markers.

### *In vivo* disposition experiment

Native proteins and ALN-modified proteins were radiolabeled with ^111^In using the bifunctional chelating agent DTPA anhydride according to the method of Hnatowich *et al*., which was described in a previous report [20,21]. In brief, DTPA-protein derivatives were synthesized in 0.1 M HEPES buffer (pH 7) and reacted with ^111^InCl_3_ to obtain ^111^In-labeled compound in 0.1 M citrate buffer (pH 5.5). The reaction mixture was applied to a PD-10 column (GE Healthcare, Little Chalfont, UK) and eluted with 0.1 M citrate buffer (pH 5.5). The derivative fractions were collected and concentrated by ultrafiltration. Each ^111^In-labeled compound was dissolved in saline, and the protein concentration was adjusted by the addition of non-radiolabeled protein to the solution. The solution of ^111^In-protein was injected into the tail vein of mice at a dose of 1 mg protein/kg. At the appropriate time after injection, blood was collected from the vena cava under ether anesthesia, and the mice were then killed. Heparin sulfate was used as an anticoagulant. Plasma was obtained from the blood by centrifugation at 5,000 g for 5 min. The liver, kidneys, spleen, heart, lungs, and bones of the lower extremity, i.e., tibiae and femurs, were collected, rinsed with saline, and weighed; urine in the bladder was also collected. The radioactivity in each sample was counted using an automatic gamma counter (1480 Wizard, Perkin-Elmer, Boston, MA, USA). The total wet bone weight was estimated as 12% of the body weight, and the radioactivity in the bone was based on activity determined in tibiae and femurs extrapolated to whole wet bone as reported previously [22].

### Calculation of area under the plasma concentration-time curve (AUC) and clearance

The ^111^In-radioactivity concentration in plasma was normalized with respect to the percentage of the dose/ml and the time courses were analyzed using the nonlinear least-squares program MULTI [23]. The tissue distribution profiles were evaluated using tissue uptake clearance (CL_tissue_) according to the integration plot analysis. The amount in a tissue at time t (Xt) and the area under the plasma concentration-time curve (AUC) from time 0 to t (AUC_0-t_) was normalized with the plasma concentration at time t (Ct), and CL_tissue_ was obtained from the slope of the plot of Xt/Ct versus AUC_0-t_/Ct [24].

### Effect of PEG-SOD-ALN on experimental bone metastasis

Experimental bone metastasis was induced by injecting 2 x 10^5^ murine B16-BL6 cells labeled with firefly luciferase gene (B16-BL6/Luc cells) [25] in 0.1 mL of RPMI into the left ventricle of C57/BL6 mice. One and three days after tumor inoculation, saline, PEG-SOD or PEG-SOD-ALN was injected into the lateral tail vein at a dose of 500 U/mouse. At 14 days after tumor inoculation, mice were injected intraperitoneally with 2.5 mg D-luciferin, anesthetized with an intraperitoneal injection of pentobarbital (50 mg/kg), and then imaging was performed using a LAS-4000 (Fujifilm, Tokyo, Japan). Then, mice were killed, and the bones of lower limbs were excised, and the luciferase activity in the tissue was measured in a luminometer (Lumat LB 9507, EG & G Berthold, Bad Wild-bad, Germany). The luciferase activity was converted to the number of the tumor cells using a regression line, as reported previously [25].

## Results

### Physicochemical characteristics of ALN-modified proteins

Table 1 summarizes the physicochemical characteristics of the synthesized ALN-modified proteins. The numbers of ALN molecules on the proteins ranged from 3 to 40. The number of ALN-modified carboxyl groups depended on the amount of reacted ALN. PEG-SOD and PEG-SOD-ALN had an average of 10 PEG molecules per SOD molecule. The isoelectric points of GR, BSA, and SOD were increased depending on the number of ALN residues, although the apparent molecular weights were barely altered by ALN modification. Unfortunately, the isoelectric points of LZM-ALN and PEG conjugates could not be determined, because of the out-of-range and neutral isoelectric point of PEG, respectively.

**Table 1:** Physicochemical characteristics of ALN-modified proteins.

Compound	Number of ALN Residues (mol/mol) ^a)^	Proportion of Modified Carboxyl Groups (%)^a)^	Number of PEG (mol/mol) ^b)^	Estimated Molecular Weight ^c)^	Isoelectric Point
GR	0	0	-	112,000	4.5
GR-ALN(4)	3.79	1.11	-	113,000	8
GR-ALN(17)	17.3	5.06	-	117,000	8.3
BSA	0	0	-	67,000	4.8
BSA-ALN (3)	2.47	2.52	-	67,800	4.8
BSA-ALN (28)	27.7	28.3	-	74,600	9.5–10.7
BSA-ALN (40)	39.7	40.5	-	77,900	>10.7
SOD	0	0	-	32,000	4.9
SOD-ALN(3)	2.99	7.88	-	32,800	4.9
SOD-ALN(32)	31.6	83.2	-	40,700	10.7
PEG(10)-SOD	0	0	9.62	82,000	N.D.
PEG(10)-SOD-ALN(4)	3.79	10	9.62	83,100	N.D.
LZM	0	0	-	14,000	11.2
LZM-ALN(5)	29.9	29.9	-	15,400	N.D.
LZM-ALN(9)	49.4	49.4	-	16,400	N.D.

^a)^ The alendronate (ALN) content was determined by the molybdenum method using ALN as a standard. The number of ALN residues was calculated using the molar ratio of ALN residues attached to the protein.

^b)^The number of PEG was estimated by the number of unreacted free amino groups, which was determined by the trinitrobenzenesulfonic acid method.

^c)^ The molecular weight of each protein derivative was estimated by considering of the number of ALN residues and PEG modifications.

N.D.; not determined

### Distribution of ^111^In-native proteins after intravenous administration

**Fig 1:**
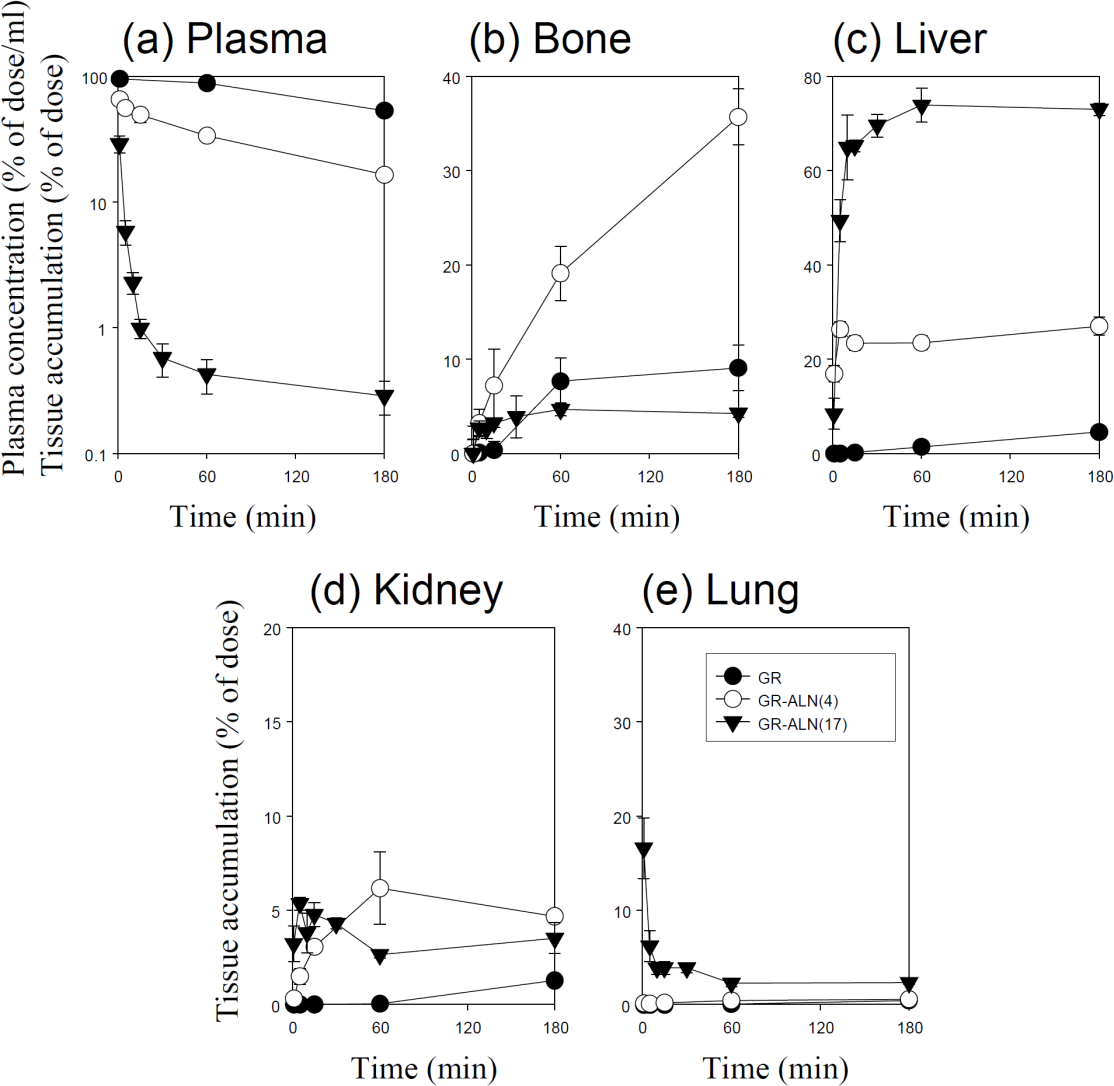
Time-courses of the plasma concentration, and bone, liver, kidney, and lung accumulation of ^111^In-labeled GR derivatives in mice after intravenous injection at a dose of 1 mg protein/kg. The total wet bone weight was estimated as 12% of the body weight, and the radioactivity in the bone was based on activity determined in tibiae and femurs extrapolated to whole wet bone as reported previously [22]. Results are expressed as the mean ± S.D. of three mice. ●: GR, ○: GR-ALN (4), ▼: GR-ALN (17).

**Fig 2:**
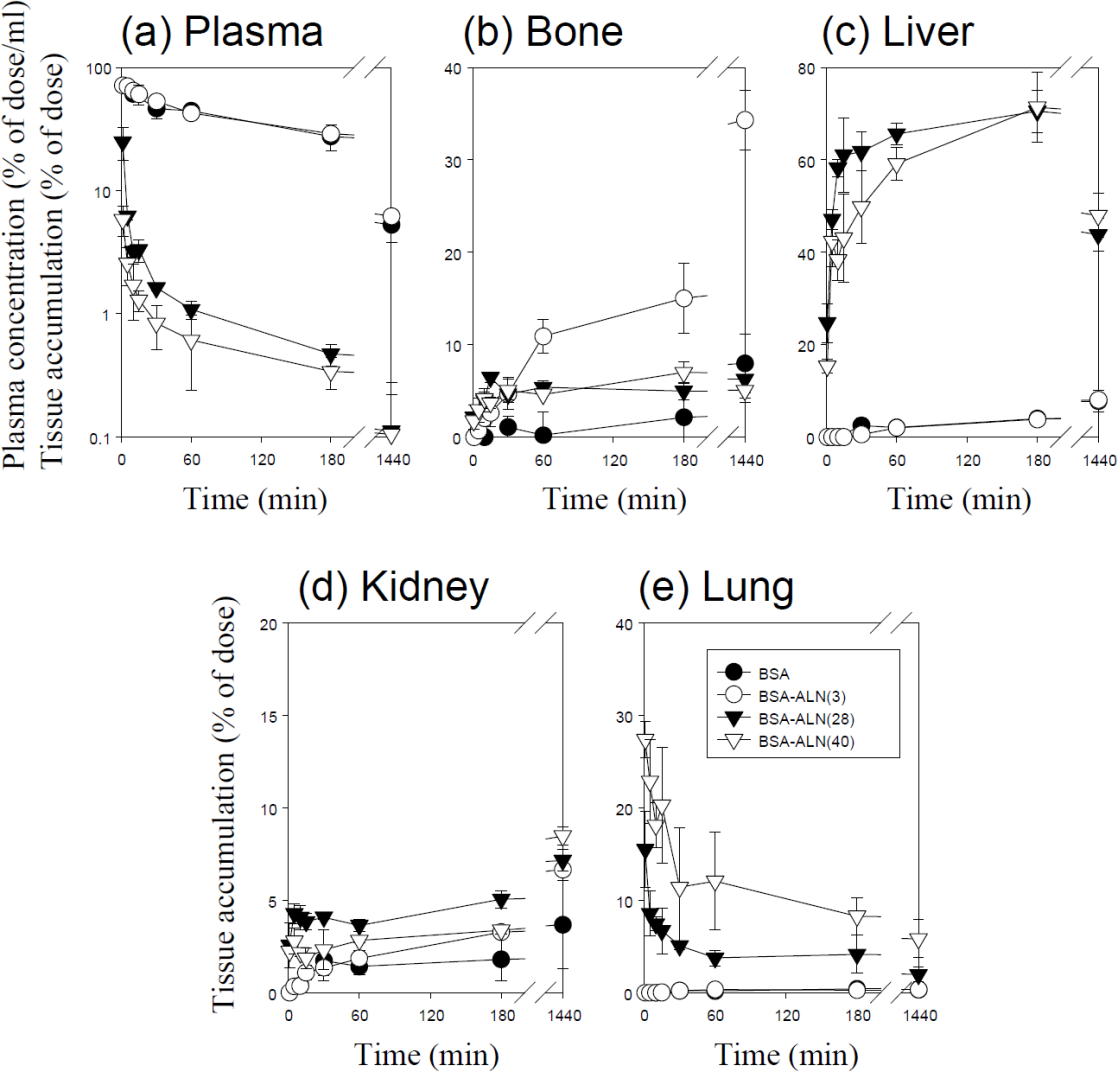
Time-courses of the plasma concentration, and bone, liver, kidney, and lung accumulation of ^111^In-labeled BSA derivatives in mice after intravenous injection at a dose of 1 mg protein/kg. The total wet bone weight was estimated as 12% of the body weight, and the radioactivity in the bone was based on activity determined in tibiae and femurs extrapolated to whole wet bone as reported previously [22].Results are expressed as the mean ± S.D. of three mice. ●: BSA, ○: BSA-ALN (3), ▼: BSA-ALN (28), ▽: BSA-ALN (40).

**Fig 3:**
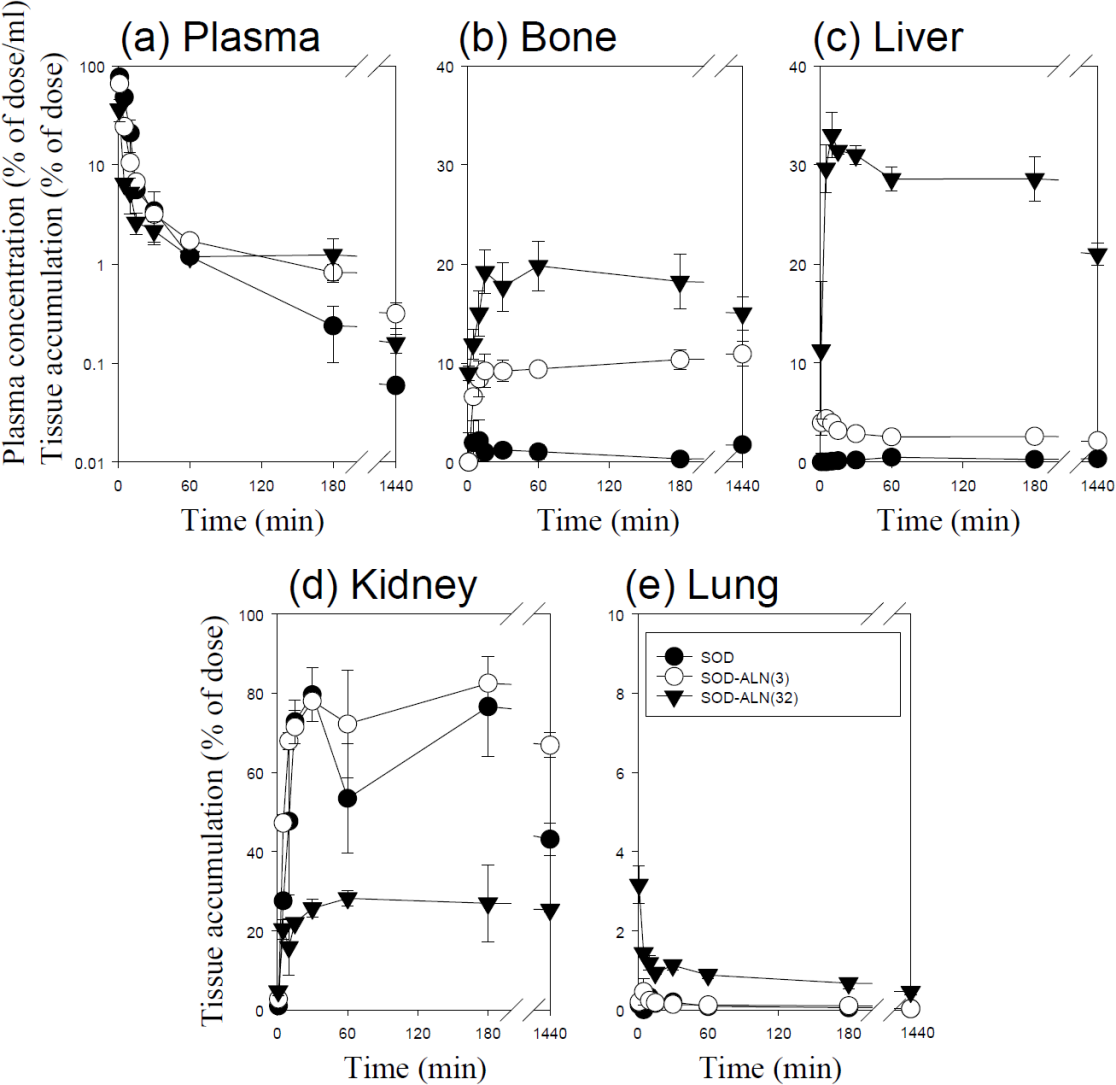
Time-courses of the plasma concentration, and bone, liver, kidney, and lung accumulation of ^111^In-labeled SOD derivatives in mice after intravenous injection at a dose of 1 mg protein/kg. The total wet bone weight was estimated as 12% of the body weight, and the radioactivity in the bone was based on activity determined in tibiae and femurs extrapolated to whole wet bone as reported previously [22].Results are expressed as the mean ± S.D. of three mice. ●: SOD, ○: SOD-ALN (3), ▼: SOD-ALN (32).

**Fig 4:**
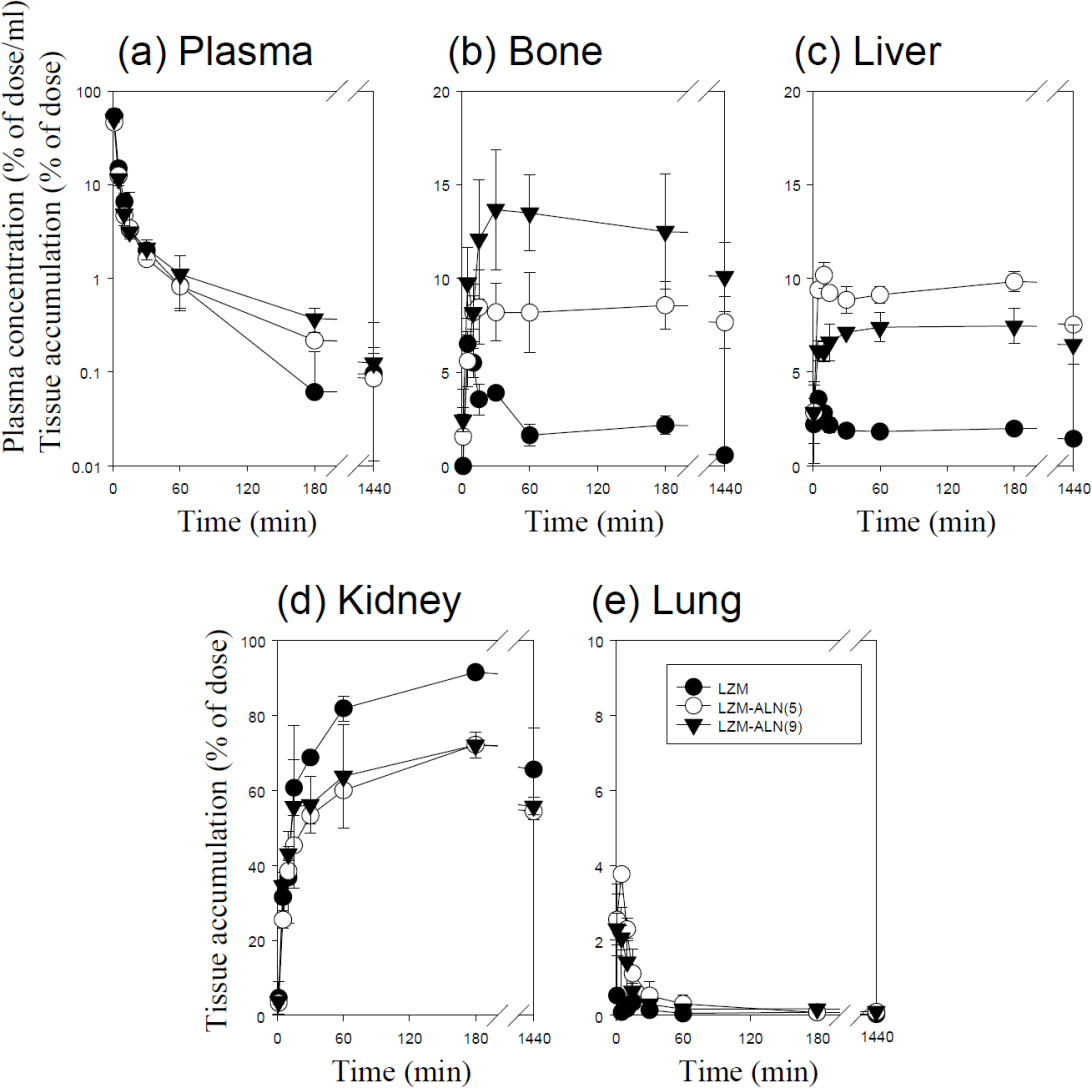
Time-courses of the plasma concentration, and bone, liver, kidney, and lung accumulation of ^111^In-labeled LZM derivatives in mice after intravenous injection at a dose of 1 mg protein/kg. The total wet bone weight was estimated as 12% of the body weight, and the radioactivity in the bone was based on activity determined in tibiae and femurs extrapolated to whole wet bone as reported previously [22].Results are expressed as the mean ± S.D. of three mice. ●: LZM, ○: LZM-ALN (5), ▼: LZM-ALN (9).

**Fig 5:**
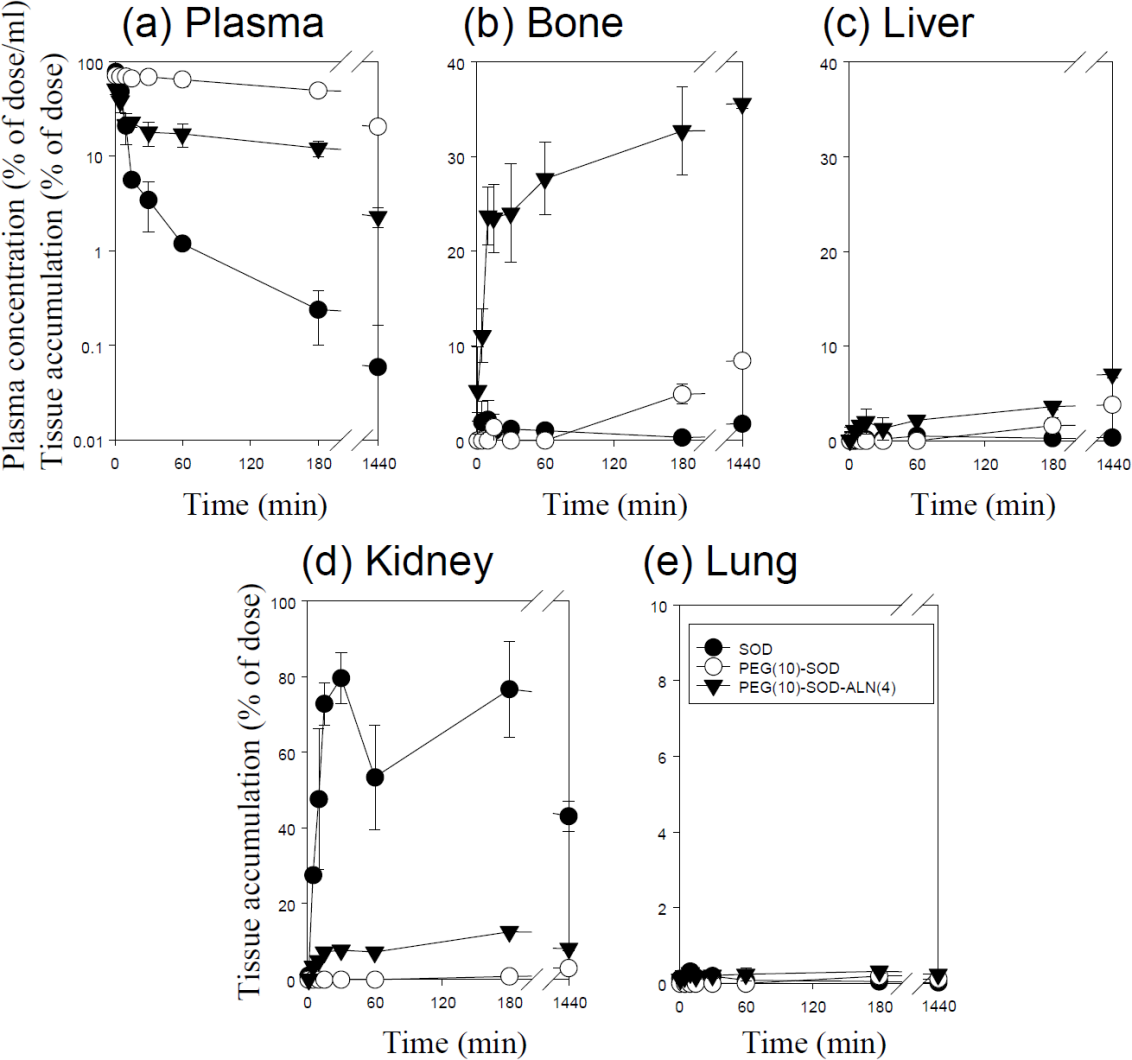
Time-courses of the plasma concentration, and bone, liver, kidney, and lung accumulation of ^111^In-labeled PEG-SOD derivatives in mice after intravenous injection at a dose of 1 mg protein/kg. The total wet bone weight was estimated as 12% of the body weight, and the radioactivity in the bone was based on activity determined in tibiae and femurs extrapolated to whole wet bone as reported previously [22]. Results are expressed as the mean ± S.D. of three mice. ●: SOD, ○: PEG(10)-SOD, ▼: PEG(10)-SOD-ALN (4).

Figs 1–5 show the time course of the plasma concentrations and the cumulative amounts of ^111^In-radioactivity in major organs after intravenous injection of ^111^In-native- and ALN-modified proteins into mice. ^111^In-LZM and ^111^In-SOD quickly disappeared from plasma, whereas the other proteins with high-molecular weights remained in the circulation for a longer period of time (Figs 3 and 4). These results are in good agreement with previous studies [26–28]. The amount of ^111^In-GR, ^111^In-BSA, ^111^In-SOD and ^111^In-LZM accumulated in bone were 9.1, 2.1, 0.3, and 2.2% of the initial dose 3 h after injection, respectively (Figs 1–4). Approximately 77 and 92% of ^111^In-radioactivity accumulated in the kidney 3 h after injection of ^111^In- SOD and LZM, respectively (Figs 3 and 4), whereas those of ^111^In-GR and ^111^In-BSA were less than 2% (Figs 1 and 2). Other tissues exhibited no significant radioactivity after injection of all ^111^In-native proteins.

### Distribution of ^111^In-ALN-modified proteins after intravenous administration

**Fig 6:**
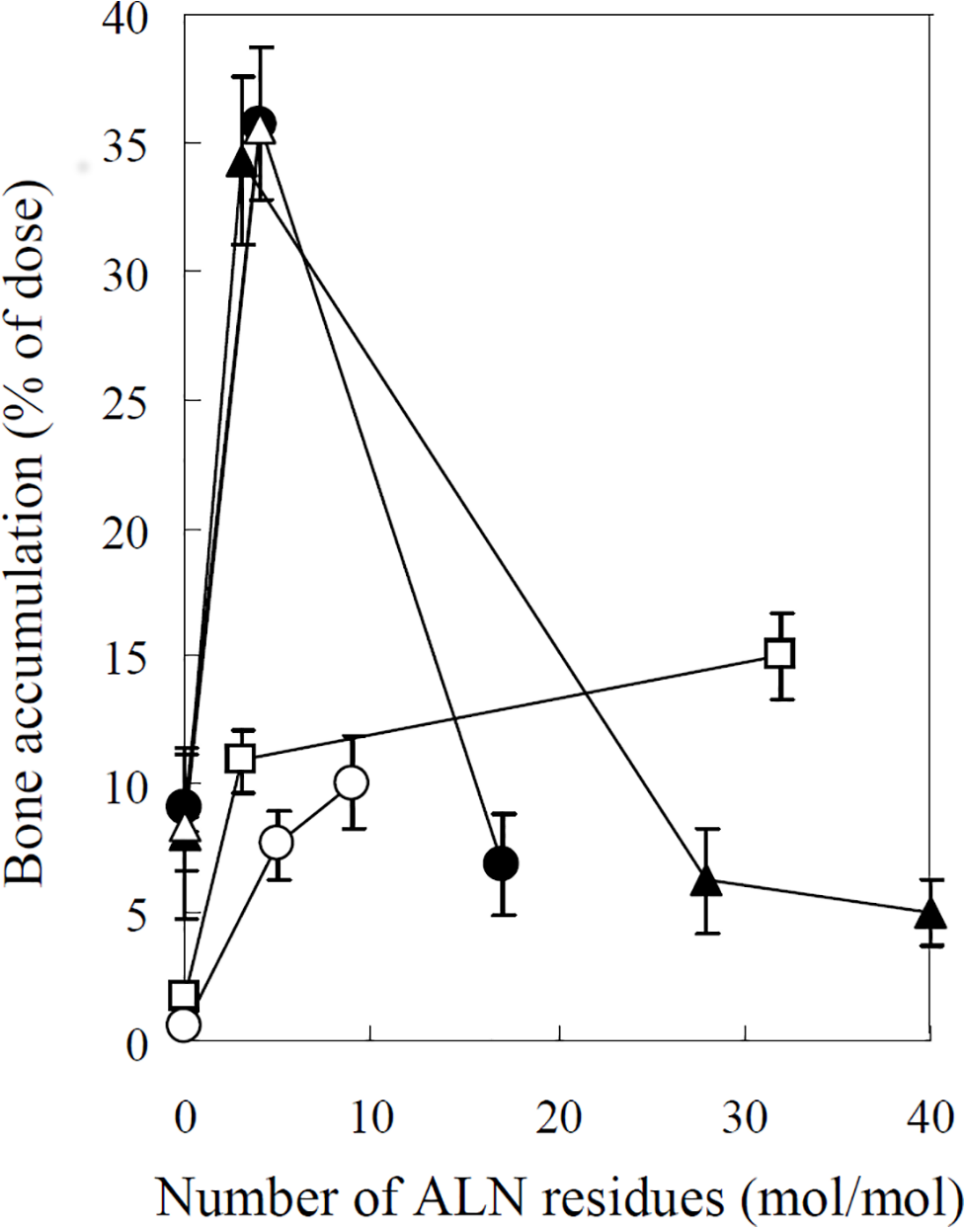
Effect of the number of ALN residues on bone accumulation of ^111^In-labeled ALN-modified proteins. ^111^In-labeled ALN-modified proteins were intravenously administered into mice at a dose of 1 mg protein/kg. The total wet bone weight was estimated as 12% of the body weight, and the radioactivity in the bone was based on activity determined in tibiae and femurs extrapolated to whole wet bone as reported previously [22]. Results are expressed as the mean ± S.D. of three mice. ●: GR-ALN, ▲: BSA-ALN, □: SOD-ALN, ○: LZM-ALN, △: PEG-SOD-ALN.

^111^In-ALN-modified proteins were taken up by the bone in various amounts, according to the degree of modification (Figs 1–5). Fig 6 summarizes the bone accumulation of ^111^In-ALN-modified proteins as a function of the number of ALN residues. The maximum amount taken up by the bone was 36, 34, 18, and 13% of the dose for ^111^In-GR-ALN(4),^111^In-BSA-ALN(3), ^111^In-SOD-ALN(32) and ^111^In-LZM-ALN(9), respectively (Figs 1–6). Although the bone accumulation of ^111^In-GR-ALN and ^111^In-BSA-ALN was greatly increased with a low degree of modification, the increase in the number of attached ALN residues accelerated accumulation in the liver, kidney, and lung, and reduced bone accumulation (Figs 1, 2 and 6). ^111^In-SOD-ALN and ^111^In-LZM-ALN accumulated in the bone depending on the number of ALN residues. However, ^111^In-radioactivity after intravenous injection of ^111^In-SOD-ALN and ^111^In-LZM-ALN was observed mainly in the kidney or liver (Figs 3 and 4 and 6). In contrast, ^111^In-PEG-SOD-ALN and ^111^In-PEG-SOD slowly disappeared from the plasma and accumulated in the bone. Approximately 36 and 8.4% of the dose were recovered in the bone 24 h after injection, respectively (Figs 5 and 6). Little radioactivity was counted in any other tissues for all ALN-modified proteins.

### Calculation of pharmacokinetic parameters

To quantitatively compare the distribution profiles of the ^111^In-native proteins and ^111^In-ALN-modified proteins, CL_total_, CL_tissue_, and AUC were calculated based on the distribution data (Table 2). ^111^In-GR and BSA had large AUCs and low total clearances, reflecting the high plasma retention after intravenous injection. The AUCs of ^111^In- SOD and ^111^In-LZM were much lower than those of ^111^In-GR and BSA, but increased depending on the molecular weight. The CL_total_ of native proteins were almost inversely correspondent with their AUC. The CL_bone_ of ^111^In-GR, ^111^In-BSA, ^111^In-SOD and ^111^In-LZM were 0.04, 0.02, 0.10, and 1.39 ml/h, respectively, which were quite low compared with their CL_total_.

**Table 2:** AUC and clearance of ^111^In-labeled ALN-modified proteins in mice after intravenous injection of 1 mg protein/kg.

Compound	AUC(h•% of dose/ml)	CL_total_ (ml/h)	CL_bone_ (ml/h)	CL_liver_ (ml/h)	CL_kidney_ (ml/h)	CL_lung_ (ml/h)	CL_urine_ (ml/h)
GR	545	0.18	0.04	0.02	0.01	0.002	0.003
GR-ALN(4)	130	0.77	0.37	0.22	0.04	0.006	0.02
GR-ALN(17)	1.91	52.4	1.37	35.8	1.87	1.37	0.38
BSA	259	0.39	0.02	0.03	0.01	0.003	0.004
BSA-ALN (3)	265	0.38	0.12	0.03	0.02	0.002	0.03
BSA-ALN (28)	1.86	53.8	2.21	32.6	2.17	3.19	0.41
BSA-ALN (40)	0.81	124	6.73	62.8	2.62	18	2.14
SOD	9.64	10.4	0.1	0.02	8.43	0.02	3.28
SOD-ALN(3)	6.49	15.4	1.51	0.45	11.8	0.02	0.75
SOD-ALN(32)	2.32	43.1	6.26	14.7	7.65	0.36	6.85
PEG(10)-SOD	207	0.48	0.12	0.03	0.02	0.002	0.002
PEG(10)-SOD-ALN(4)	30.8	3.25	0.94	0.11	0.38	0.01	0.06
LZM	4.18	24	1.39	0.65	9.5	0.38	2.99
LZM-ALN(5)	3.48	28.7	2.55	3.06	12.1	0.59	2.43
LZM-ALN(9)	3.5	28.6	2.35	1.76	13.1	0.35	2.54

ALN modification increased the CL_bone_ of all the proteins, without significantly changing the tissue uptake clearance in any other organ, except for the liver, kidneys, and lungs. The maximum CL_bone_ of ^111^In-GR-ALN, ^111^In-BSA-ALN, ^111^In-SOD-ALN, and ^111^In-LZM-ALN were 1.37, 6.73, 6.26, and 2.55 ml/hr, respectively, which were much higher than those of native protein. The CL_total_ of ^111^In-ALN-modified proteins were proportional to the degree of modification, and CL_liver_, CL_renal_, or CL_lung_ accounted for most of the CL_total_ in proteins with a high degree of modification.

Among the series of SOD derivatives, PEG-SOD had the highest AUC. The AUC of ^111^In-PEG-SOD-ALN was much higher than that of ^111^In-SOD-ALN and ^111^In-SOD. Additionally, the CL_liver_ and CL_kidney_ of ^111^In-PEG-SOD-ALN were much lower than those of ^111^In-SOD-ALN and ^111^In-SOD. In addition, ^111^In-PEG-SOD-ALN showed the highest CL_bone_ among the SOD derivatives. The uptake rate in the bone of ^111^In-PEG-SOD-ALN was 94-fold greater than that of ^111^In-SOD.

### Inhibition of bone metastasis of melanoma cells by PEG-SOD-ALN

**Fig 7:**
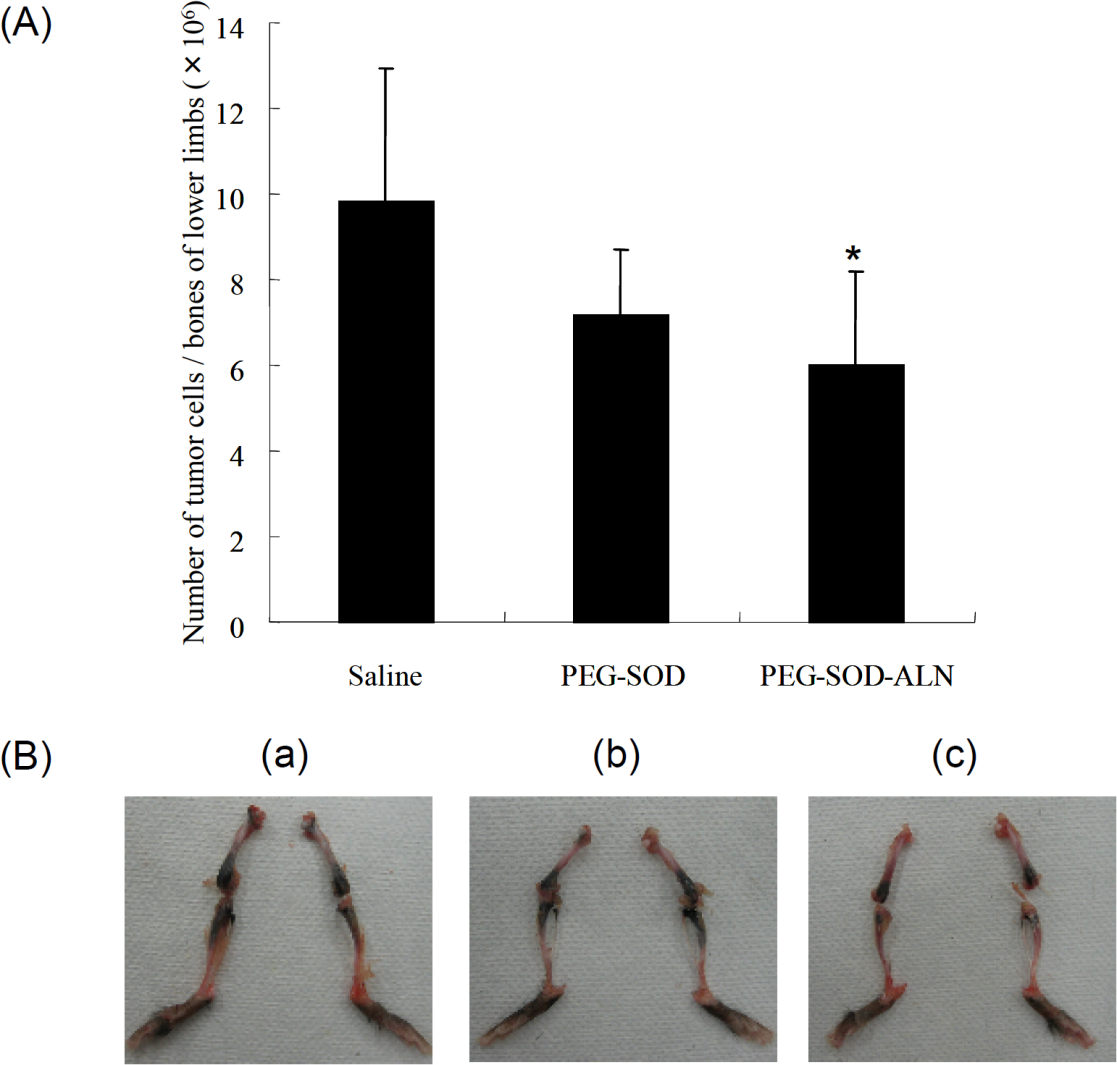
Effect of SOD derivatives on the growth of metastatic tumor cells in the bones of lower limbs after its administrations 1 and 3 days after tumor injection. (A) Number of B16BL6/Luc cells in the bones of lower limb of mice administered with PEG-SOD or PEG-SOD-ALN. Mice were sacrificed at 14 days after tumor inoculation. Results are expressed as the mean ± SD of 5–6 mice. *, a statistically significant difference compared with the saline group (P < 0.05). (B) Macroscopic observation of bone metastasis in the mouse lower limb at 14 days after inoculation of B16BL6/Luc cells (2×10^5^ cells/mouse) into the mouse left ventricle. (a) Saline, (b) PEG-SOD, (c) PEG-SOD-ALN.

Fig 7A shows the number of B16–BL6/Luc cells in the tibiae and femurs of the bone metastasis mouse model. Treatment with PEG-SOD-ALN significantly reduced the number of tumor cells in the bone of the mice. In marked contrast, PEG-SOD had no statistically significant effects on the number of tumor cells in the bones.

Fig 7B shows the macroscopic observation of B16–BL6/Luc cells in the tibiae and femurs of the bone metastasis mouse model. PEG-SOD-ALN effectively suppressed the metastatic colonies in the tibiae and femurs of the bone metastasis mouse model. S1 Fig shows the *in vivo* imaging on the distribution of the tumor cells in live mice monitored by the luciferase activity. Similarly, the growth of metastatic tumor cells was effectively inhibited by PEG-SOD-ALN. These data strongly supported the quantitative results.

### Statistical analysis

Statistical comparisons with the vehicle control group were performed using Dunnett's test at a significance level of *p* < 0.05.

## Discussion

To establish a rational molecular design for BP-modified proteins to efficiently target the bone *in vivo*, we performed a systemic pharmacokinetic study using a series of ALN-modified proteins with various molecular weights and degrees of modification. The pharmacokinetic study showed that the bone uptake clearance of ALN-modified proteins was closely related to the degree of ALN modification. In a preliminary experiment, we examined the stability of BSA-ALN(3) in mouse plasma by measuring free ALN using HPLC. No free ALN was detected in the sample 3 h after incubation at 37°C (data not shown), indicating that the linkage of ALN with proteins was stable in plasma. Therefore, the high bone uptake clearance of ALN-modified proteins could be explained by the high affinity of ALN to hydroxylapatite, the major component of bone [6–8,11,12]. However, ALN modification did not change the high renal clearance of SOD and LZM, the two low-molecular weight proteins, and hardly increased the bone accumulation compared with GR and BSA, the high-molecular weight proteins with long blood circulation. These results suggest that the affinity of ALN to bone is not sufficient for the effective bone targeting of proteins with high total-body clearance. The greater bone distribution of PEG-SOD-ALN could be the consequence of reduced renal clearance, affording a greater opportunity to interact with bone, because it was reported that PEG modification increases the plasma retention of proteins *in vivo* [16, 29, 30]. Although there was a concern that the interaction of ALN of the ALN-modified proteins with bone could be sterically prevented by the bulky PEG molecules [13], the pharmacokinetic analysis in the present study indicates that PEG modification barely reduces the affinity of SOD-ALN to bone. Thus, the combination of PEG and ALN modification is a promising approach for efficient bone targeting of proteins with high total-body clearance.

The bone accumulation of GR-ALN and BSA-ALN, the two high-molecular weight proteins, was almost inversely correlated with the number of ALN residues. In general, the electric charge of proteins also plays an important role in the tissue distribution of protein derivatives [5, 31–33]. It was reported that cationic proteins, such as amidated proteins, are rapidly accumulated in the liver after intravenous injection [32,33]. In the present study, ALN modification increased the positive charge depending on the number of ALN residues, likely due to the fact that the carboxyl residues of the proteins are modified. These results suggest that the greater hepatic distribution of GR-ALN and BSA-ALN with large number of ALN moieties is due to the electrostatic interaction between the negatively charged cell surface and the increased positive charge of GR and BSA [32, 33]. Furthermore, it was also reported that high concentrations of BPs form insoluble complexes with calcium or insoluble aggregates in the blood [34]. Therefore, the large number of ALN residues on GR-ALN and BSA-ALN may also enhance the aggregation of GR-ALN or BSA-ALN in the blood through interactions with calcium in the blood, thereby enhancing uptake by the reticuloendothelial system in the liver and accumulation in the pulmonary capillary. These two effects could be interrelated in the tissue distribution, affecting the fate of GR-ALN and BSA-ALN with a large number of ALN moieties. Although the calculation of bone uptake clearance revealed that the affinity of ALN-modified proteins to bone was proportional to the degree of modification, the results of the tissue distribution suggest that approximately 3–4 ALN/protein is the optimal degree of modification for bone targeting of these proteins.

It was reported that bones possess a membrane consisting of lining cells, which function as a marrow-blood barrier. Thus, the accessibility of exogenous large substances to the bone surface is extremely limited [7, 35]. In contrast, it was also reported that exogenous macromolecules, which are approximately 60 nm in diameter, accumulated within myeloid bone marrow interstitial space [36]. Thus, there have been several controversies concerning the vascular permeability of bone marrow. In the present study, ALN-modified proteins showed high bone uptake clearance (0.12–6.73 ml/h) compared with unmodified proteins (0.02–1.39 ml/h). Previous studies reported that the bone plasma flow rate was approximately 10.2 ml/h for a 22 g mouse [37]. These results indicate that the bone uptake of proteins is not limited by the marrow-blood barrier. In bone metastasis, where tumor cells cause vascular endothelial cell junctions to loosen and increase vascular permeability [38], the transport of ALN-modified proteins through the marrow-blood barrier would be considered relatively easy. In addition, it was reported that BPs have a high affinity to hydroxyapatite at areas of increased bone turnover, such as bone metastasis [12].

The inhibitory effects on bone metastasis treated with PEG-SOD-ALN may be associated with these results, together with those on pharmacokinetics studies in the present study. Because reactive oxygen species (ROS) play important roles in a series of steps of tumor metastasis and the tumor metastasis is effectively prevented by targeting of ROS scavenging SOD and catalase [39], the inhibitory effect on bone metastasis treated with PEG-SOD-ALN could be explained by efficient ROS elimination in the bone. Although further studies are needed to elucidate the detailed mechanism underlying this inhibition, these results suggest that our bone targeting strategy using BP is a promising approach for the treatment of bone metastasis.

## Conclusions

The efficient bone targeting of GR and BSA, two high-molecular weight proteins with long blood circulation, was successfully achieved by ALN modification; however, decreased bone distribution was observed with a high degree of modification. These results indicate that approximately 3–4 ALN/protein is an optimal degree of modification for bone targeting of proteins. In contrast, low-molecular-weight proteins, such as SOD and LZM with a high total-body clearance, need both modification with 3–4 ALN/protein and PEG for efficient delivery to bone. Thus, the present study has provided useful basic information for therapeutic strategies and the molecular design of BP-modified proteins for use as therapeutic agents and drug carriers for bone targeting *in vivo*.

## Supporting Information

S1 Fig Effect of SOD derivatives on the growth of metastatic tumor cells in the bones of lower limbs after its administrations 1 and 3 days after inoculation of B16BL6/Luc cells (2×10^5^ cells/mouse) into the mouse left ventricle. Visualization of firefly luciferase gene expression in the lower limb at 14 days after inoculation. (a) Saline, (b) PEG-SOD, (c) PEG-SOD-ALN.
(TIF)
