# Supplementary figures and images for: Molecular Design of Bisphosphonate-Modified Proteins for Efficient Bone Targeting In Vivo
Source: PLoS One. 2015 Aug 19;10(8):e0135966. doi: 10.1371/journal.pone.0135966 (PMC4545940; doi:10.1371/journal.pone.0135966)

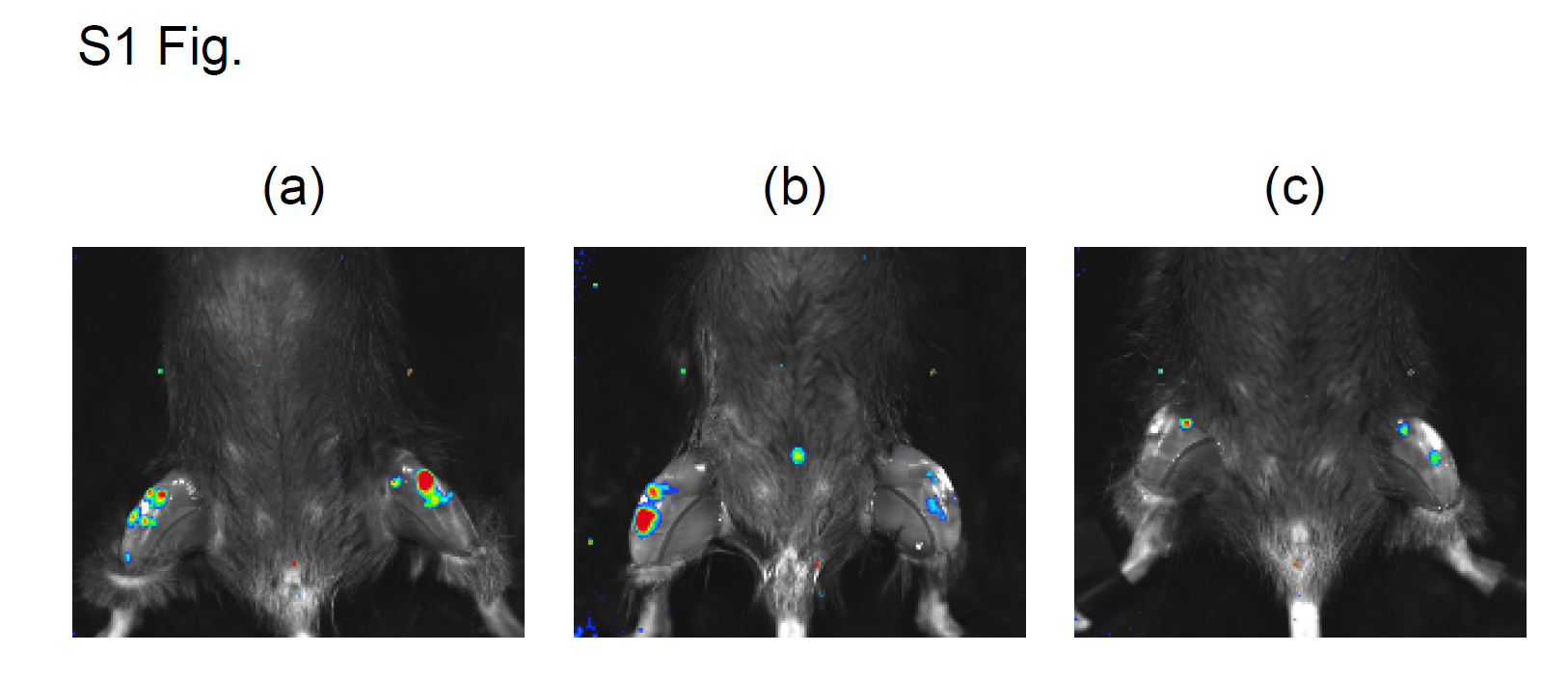

Supplement: S1 Fig — Visualization of firefly luciferase gene expression in the lower limb at 14 days after inoculation. (a) Saline, (b) PEG-SOD, (c) PEG-SOD-ALN. (TIF) [file pone.0135966.s001.tif]
